# Crosslinked Structure of Polyacrylic Acid Affects Pulmonary Fibrogenicity in Rats

**DOI:** 10.3390/ijms232213870

**Published:** 2022-11-10

**Authors:** Taisuke Tomonaga, Chinatsu Nishida, Hiroto Izumi, Naoki Kawai, Ke-Yong Wang, Hidenori Higashi, Jun-Ichi Takeshita, Ryohei Ono, Kazuki Sumiya, Shota Fujii, Yuki Hata, Kazuo Sakurai, Toshiki Morimoto, Yasuyuki Higashi, Kei Yamasaki, Kazuhiro Yatera, Yasuo Morimoto

**Affiliations:** 1 Department of Occupational Pneumology, Institute of Industrial Ecological Sciences, University of Occupational and Environmental Health, Japan 1-1 Iseigaoka, Yahata Nishi-ku, Kitakyushu 807-8555, Japan; 2 Department of Respiratory Medicine, University of Occupational and Environmental Health, Japan 1-1 Iseigaoka, Yahata Nishi-ku, Kitakyushu 807-8555, Japan; 3 Shared-Use Research Center, School of Medicine, University of Occupational and Environmental Health, Japan 1-1 Iseigaoka, Yahata Nishi-ku, Kitakyushu 807-8555, Japan; 4 Department of Environmental Health Engineering, Institute of Industrial Ecological Sciences, University of Occupational and Environmental Health, Japan 1-1 Iseigaoka, Yahata Nishi-ku, Kitakyushu 807-8555, Japan; 5 Research Institute of Science for Safety and Sustainability, National Institute of Advanced Industrial Science and Technology (AIST), Tsukuba, Japan 16-1 Onogawa, Tsukuba 305-8569, Japan; 6 Department of Chemistry and Biochemistry, The University of Kitakyushu, 1-1, Hibikino, Wakamatsu-ku, Kitakyushu 808-0135, Japan

**Keywords:** polyacrylic acid (PAA), organic chemicals, crosslinking, pulmonary toxicity

## Abstract

We conducted intratracheal instillations of polyacrylic acid (PAA) with crosslinking and non-crosslinking into rats in order to examine what kinds of physicochemical characteristics of acrylic-acid-based polymers affect responses in the lung. F344 rats were intratracheally exposed to similar molecular weights of crosslinked PAA (CL-PAA) (degree of crosslinking: ~0.1%) and non-crosslinked PAA (Non-CL-PAA) at low and high doses. Rats were sacrificed at 3 days, 1 week, 1 month, 3 months, and 6 months post-exposure. Both PAAs caused increases in neutrophil influx, cytokine-induced neutrophil chemoattractants (CINC) in the bronchoalveolar lavage fluid (BALF), and heme oxygenase-1 (HO-1) in the lung tissue from 3 days to 6 months following instillation. The release of lactate dehydrogenase (LDH) activity in the BALF was higher in the CL-PAA-exposed groups. Histopathological findings of the lungs demonstrated that the extensive fibrotic changes caused by CL-PAA were also greater than those in exposure to the Non-CL- PAA during the observation period. CL-PAA has more fibrogenicity of the lung, suggesting that crosslinking may be one of the physicochemical characteristic factors of PAA-induced lung disorder.

## 1. Introduction

Inorganic chemicals such as asbestos and crystalline silica are known to cause fibrosis-based lung disorders such as pneumoconiosis, while organic chemicals are known to cause allergic diseases such as bronchial asthma and hypersensitivity pneumonitis, but are not considered to cause lung fibrosis directly. In recent years, however, it has been reported that organic chemicals induce fibrosis following inflammation. In Korea, general consumers exposed to disinfectants used in humidifiers developed progressive interstitial pneumonia, which became a social problem [1,2], and in the United States, e-cigarette users suffered from acute respiratory failure [3,4]. In Japan, progressive lung disorder was observed in workers who handled several polyacrylic acids (PAA), and the Ministry of Health, Labour and Welfare (MHLW) considered this lung disorder important and issued a notice [5,6,7].

PAA is a white powder that is composed of acrylic acid monomers that are repeatedly linked together to form a so-called polymer structure, and by using a crosslinking agent to induce covalent crosslinking of multiple linear molecules, the polymer is made more polymeric, forming a complex three-dimensional structure [8]. Since PAA is an organic chemical that has water absorbency and thickening properties when dissolved in water, it is used in various products in daily use—from diapers, shampoos, and cosmetics to food additives—and is a highly safe substance that is widely used in general society [9]. In Japan, however, inflammation and fibrosis of the interstitium around the airways have been reported in workers handling PAAs that are water-absorbing crosslinked polymer compounds [5,6,7]. The progression of fibrosis is more rapid than that of lung disorder caused by asbestos or crystalline silica, which are known as representative inorganic chemicals with fibrogenicity. Elucidation of its pathogenesis is urgently needed.

Several animal exposure studies using PAA have confirmed that its pathogenesis is lung disorder, as in humans [7,10,11]. Rat intratracheal instillation and inhalation exposure studies of PAA have reported neutrophilic lung inflammation, lung injury, and subsequent rapid fibrosis [7,10,11]; however, it has not yet been elucidated as to what physicochemical properties of PAA affect the physiopathology in the lung.

We previously reported in an intratracheal instillation in rats [12] that PAA with high molecular weights causes more lung disorders than PAA with low molecular weights. It is thought that the thickening property changes with increasing molecular weight, resulting in an advance in the inflammogenicity and fibrogenicity of PAA. The crosslinking structure is also considered to be another major factor that affects the thickening property. In the present study, an intratracheal instillation was performed on rats using crosslinked PAA (CL-PAA) and non-crosslinked PAA (Non-CL-PAA) at the same molecular weight level in order to evaluate whether or not crosslinking structures of PAA affect physiopathology in lung.

## 2. Results

### 2.1. Relative Lung Weights

**Figure 1:**
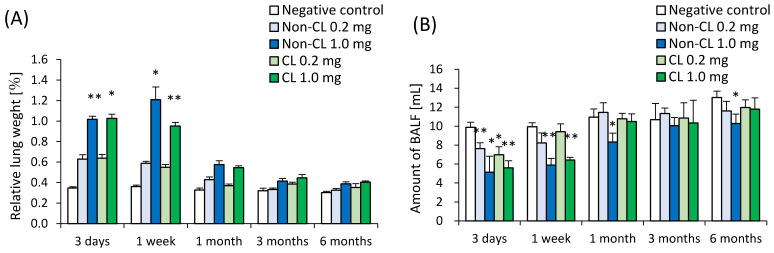
Relative lung weight after the instillation, and amount of BALF in the dissection. (**A**) Relative weight of the entire lung was calculated as a ratio of total lung weight (g) to body weight for each rat in the Non-CL-PAA and CL-PAA exposure groups. (**B**) The amounts of BALF were obtained during dissection at each time after exposure. The relative lung weight was generally significantly heavier in the exposed groups compared with the control group in a dose-dependent manner throughout the observation period. The amounts of BALF were also significantly lower in the exposed groups compared with the control group in a dose-dependent manner throughout the observation period. Data are presented as mean ± SD. (* *p* < 0.05, ** *p* < 0.01). An average of negative controls in Non-CL-PAA and CL-PAA experiments at each time point showed a single bar of negative control.

**Figure 2:**
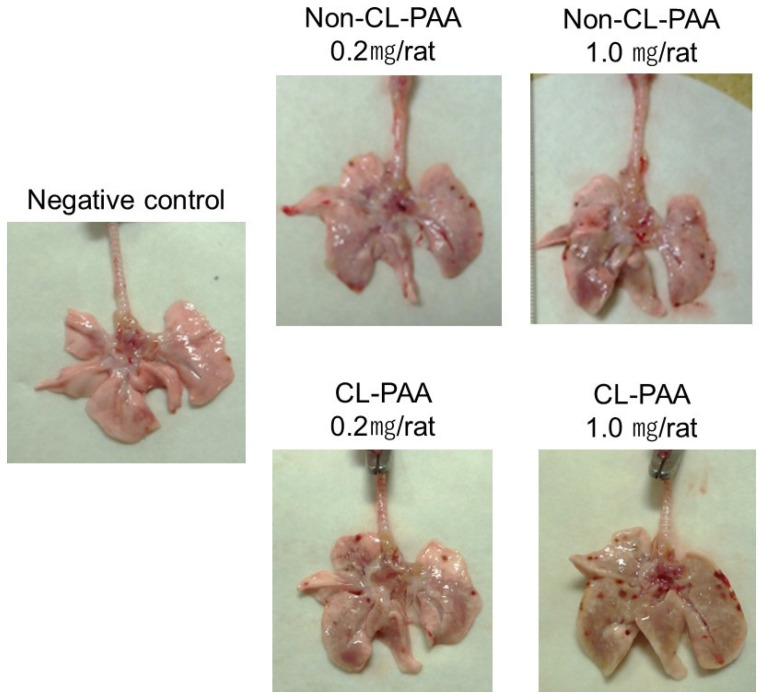
Macro findings 3 days after the instillation. The lungs in the exposed groups showed swelling at 3 days after intratracheal instillation. In the 1.0 mg CL-PAA-exposed group, the surface of the lungs was rigid, and it was difficult to expand the lungs when BALF was obtained.

Relative lung weight (lung weight/body weight) increased dose-dependently at each observation point in both the Non-CL-PAA and CL-PAA exposure groups (Figure 1A). The amounts of BALF were significantly lower in both the Non-CL-PAA and CL-PAA exposure groups compared with the control group in a dose-dependent manner throughout the observation period (Figure 1B). The CL-PAA-exposed lungs, especially the 1.0 mg of CL-PAA-exposed lungs, were more rigid, and it was difficult to expand the lungs when obtaining the bronchoalveolar lavage fluid (BALF) (Figure 2).

### 2.2. Cell Analysis and Cell Injury Markers in Bronchoalveolar Lavage Fluid (BALF)

**Figure 3:**
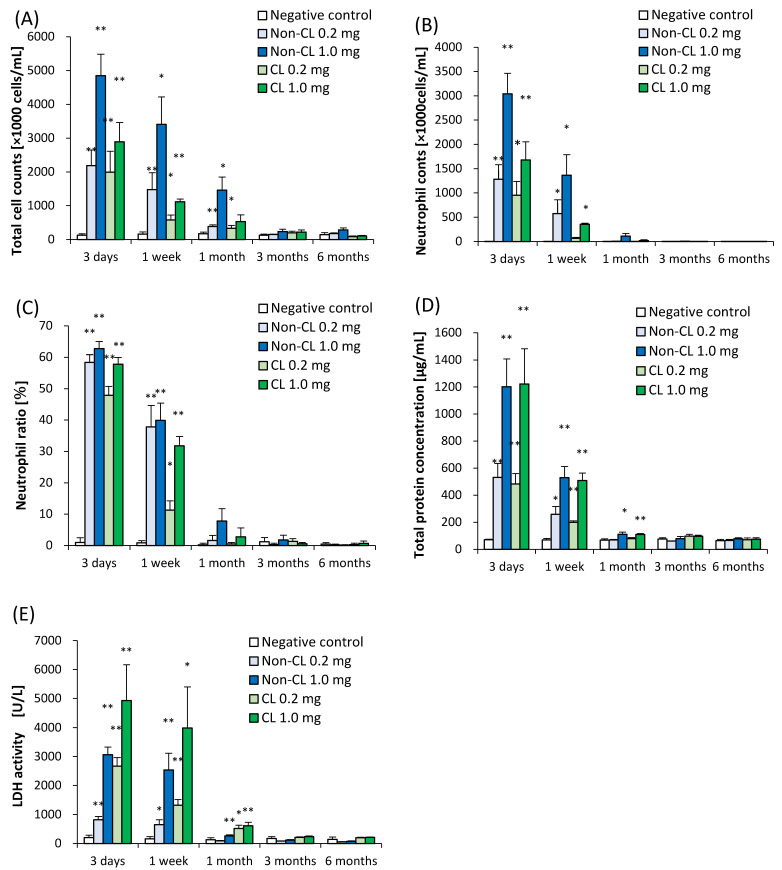
Analysis of cell number, released LDH activity, and total protein in BALF following intratracheal instillation of Non-CL-PAA and CL-PAA. (**A**) Total cell number in BALF. (**B**) Neutrophil count in BALF. (**C**) Percentage of neutrophils in BALF. (**D**) Concentration of total protein in BALF. (**E**) Released LDH activity in BALF. Inflammatory cell counts, released LDH activity, and concentration of total protein in BALF in all of the exposed groups were higher than those in the control groups in a dose-dependent manner at 3 days to 1 month after exposure. Inflammatory cells in BALF were more increased in the Non-CL-PAA than in the CL-PAA exposure groups, while released LDH activity in BALF was higher in the CL-PAA. Data are presented as mean ± SD (* *p* < 0.05, ** *p* < 0.01). An average of negative controls in the Non-CL-PAA and CL-PAA experiments at each time point showed a single bar of negative control.

Figure 3 shows the results of inflammatory cell counts, lactate dehydrogenase (LDH) activity, and total protein in BALF. There were statistically significant increases in the number of total cells, neutrophils, and percentage of neutrophils in all of the exposure groups from 3 days to 1 month after exposure compared with each control group (Figure 3A–C). The results of LDH activity and total protein in all of the exposure groups also showed statistical tendencies to increase from 3 days to 1 month after exposure compared with the control group (Figure 3D,E). There was a greater increase in inflammatory cells in the BALF in the Non-CL-PAA-exposed groups than in the CL-PAA, while the release of LDH activity in the BALF was higher in the CL-PAA-exposed groups.

### 2.3. Concentration of Cytokine-Induced Neutrophil Chemoattractant (CINC) in BALF and Concentration of Heme Oxygenase (HO)-1 in Lung Tissue

**Figure 4:**
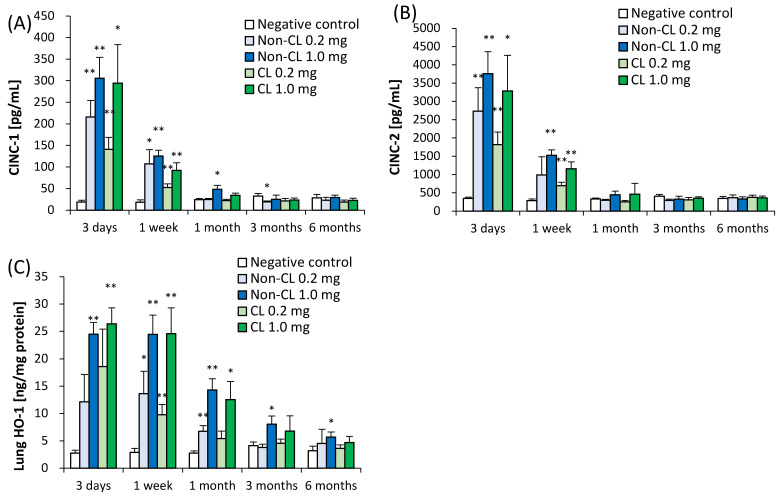
Analysis of cytokines in BALF and HO-1 in lung tissue after intratracheal instillation. (**A**) CINC-1 concentration in BALF. (**B**) CINC-2 concentration in BALF. (**C**) HO-1 concentration in lung tissue. CINC-1 and CINC-2 in BALF showed a tendency to increase in all exposure groups from 3 days to 1 week. HO-1 in lung tissue showed an increase in a dose-dependent manner from 3 days to 1 month, and a tendency to increase in the 1.0 mg exposure group of both PAAs until 6 months after exposure. Data are presented as mean ± SD. (* *p* < 0.05 and ** *p* < 0.01 indicate that the values are significantly higher than the control group.) An average of negative controls in the Non-CL-PAA and CL-PAA experiments at each time point showed a single bar of negative control.

Figure 4 shows the concentrations of CINC-1 and CINC-2 in the BALF and HO-1 in lung tissue following the intratracheal instillation of Non-CL-PAA and CL-PAA. The CINC-1 and CINC-2 in the BALF showed an upward trend in all of the exposure groups from 3 days to 1 week. The HO-1 in the lung tissue showed an increase in a dose-dependent manner from 3 days to 1 month, and there was a tendency to increase in the 1.0 mg exposure group of both PAAs until 6 months after exposure. These results indicate that Non-CL-PAA and CL-PAA induced transient increases of chemokines and lung injury from an acute phase after exposure, except for an oxidative stress marker.

### 2.4. Histopathological Features in the Lung

**Figure 5:**
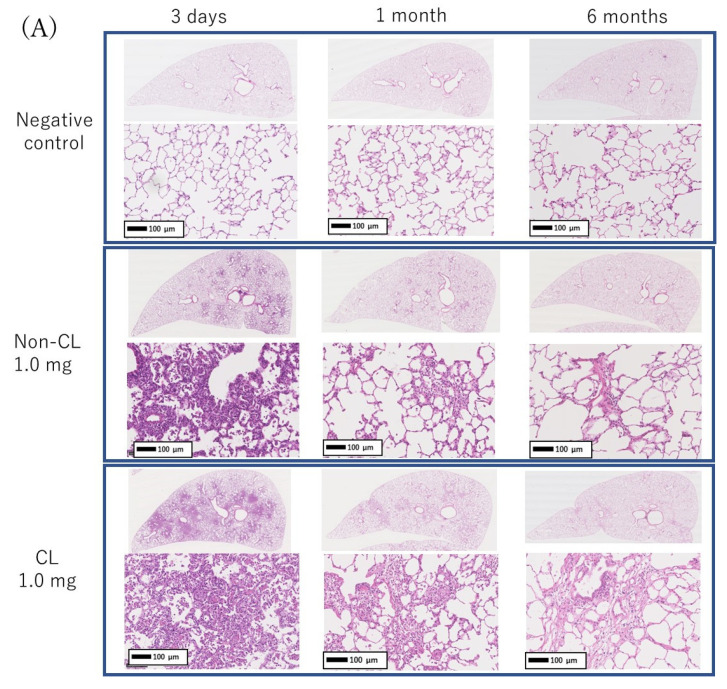

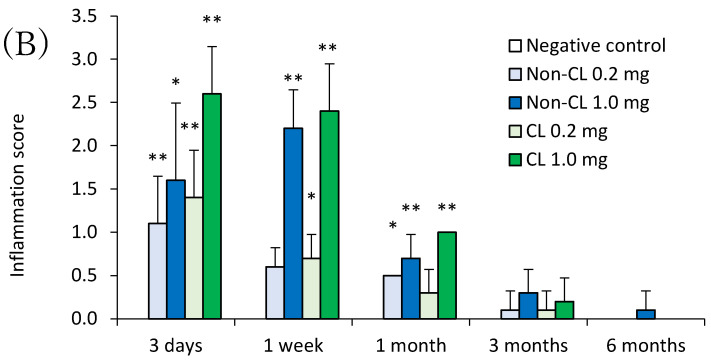
Histopathological findings of HE staining in lung exposed to Non-CL-PAA and CL-PAA. (**A**) Representative histopathological findings in the lung at 3 days, 1 month, and 6 months after the instillation of Non-CL-PAA and CL-PAA. (**B**) Inflammation score in histopathological findings of lung. Inflammatory cell infiltrations into the alveoli, mainly neutrophils, were remarkable in the lung in a dose-dependent manner at 3 days after exposure to both Non-CL-PAA and CL-PAA. Fibroinflammatory changes were observed from 3 days to 1 week after exposure. An average of negative controls in Non-CL-PAA and CL-PAA experiments at each time point showed a single bar of negative control. Data are presented as mean ± SD. (* *p* < 0.05 and ** *p* < 0.01 indicate that the values are significantly higher than the control group.) An average of negative controls in the Non-CL-PAA and CL-PAA experiments at each time point showed a single bar of negative control.

**Figure 6:**
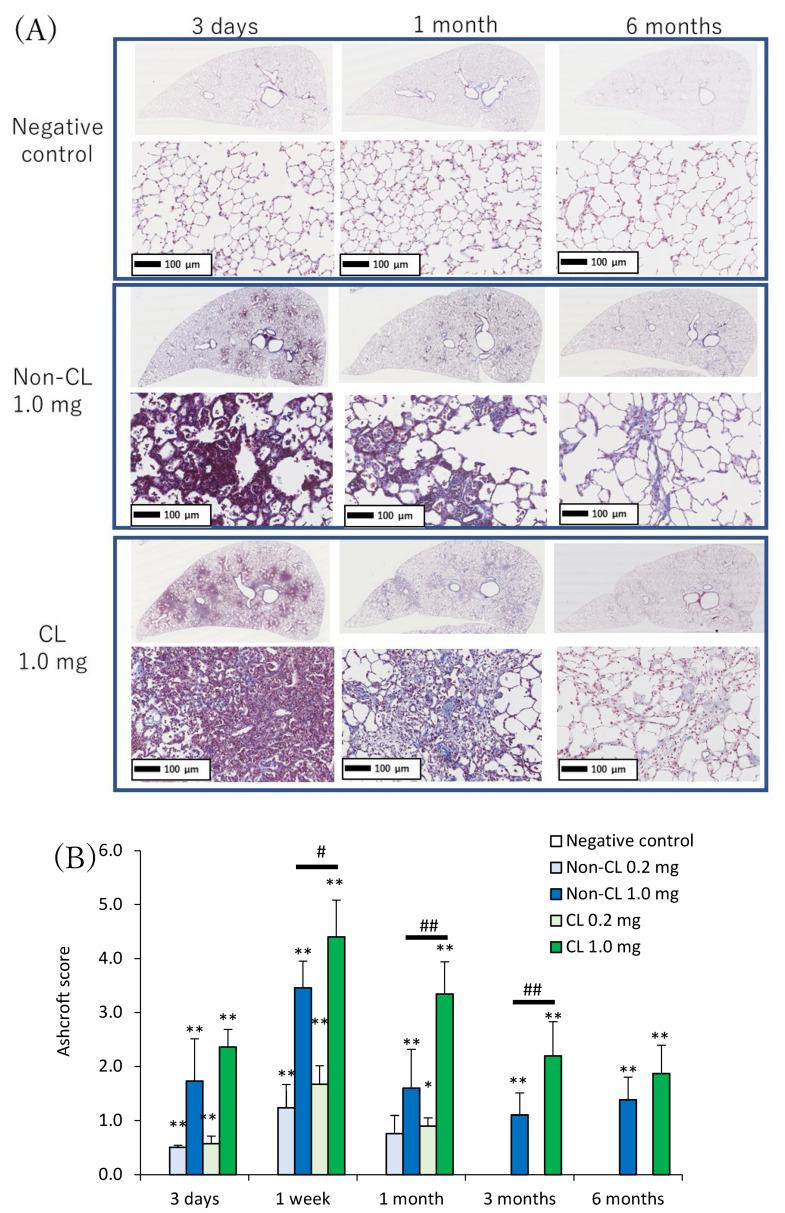
Histopathological findings of MT staining in lung exposed to Non-CL-PAA and CL-PAA. (**A**) Representative histopathological findings in the lung at 3 days, 1 month, and 6 months after the instillation of Non-CL-PAA and CL-PAA. (**B**) Ashcroft score, which indicates fibrosis score, in histopathological findings of lung. Fibrosis was also observed in the 1.0 mg exposure groups of both Non-CL-PAA and CL-PAA, whereas fibrosis was more widespread in the 1.0 mg exposure group of CL-PA. Evaluation by the Ashcroft score showed a dose-dependent increase in the scores in the exposure groups of both Non-CL-PAA and CL-PAA. There were higher scores in the 1.0 mg exposure group of CL-PAA than in that of Non-CL-PAA. An average of negative controls in Non-CL-PAA and CL-PAA experiments at each time point showed a single bar of negative control. Data are presented as mean ± SD. (* *p* < 0.05 and ** *p* < 0.01 indicate that the values are significantly higher than the control group; # *p* < 0.05 and ## *p* < 0.01 indicate that the values are significantly higher than in the Non-CL-PAA 1.0 mg exposure group). An average of negative controls in the Non-CL-PAA and CL-PAA experiments at each time point showed a single bar of negative control.

Representative histopathological findings in the lung at 3 days, 1 month, and 6 months after the instillation of Non-CL-PAA and CL-PAA are shown in Figure 5A. Inflammatory cell infiltrations into the alveoli, mainly neutrophils, were remarkable in the lung in a dose-dependent manner at 3 days after exposure to both Non-CL-PAA and the CL-PAA. Fibroinflammatory changes were observed from 3 days to 1 week after exposure, indicating a high degree of lung injury. In the 1.0 mg exposure group of CL-PAA especially, there were stronger clusters of inflammatory cells than in Non-CL-PAA. Evaluation by the inflammation score showed a dose-dependent increase in the score in the exposure to both Non-CL-PAA and CL-PAA, and there were tendencies of higher scores in the 1.0 mg exposure group of CL-PAA than in that of Non-CL-PAA (Figure 5B). Similar to the spread of inflammatory cell infiltration, fibrosis was also observed in the 1.0 mg exposure group of both Non-CL-PAA and CL-PAA, whereas fibrosis was more widespread in the 1.0 mg exposure group of CL-PAA (Figure 6A). Evaluation by the Ashcroft score showed a dose-dependent increase in the score of the exposure to both Non-CL-PAA and CL-PAA. There were higher scores in the 1.0 mg exposure group of CL-PAA than of Non-CL-PAA (Figure 6B).

### 2.5. The Relationship between Lung Fibrosis and Lung Injury

**Figure 7:**
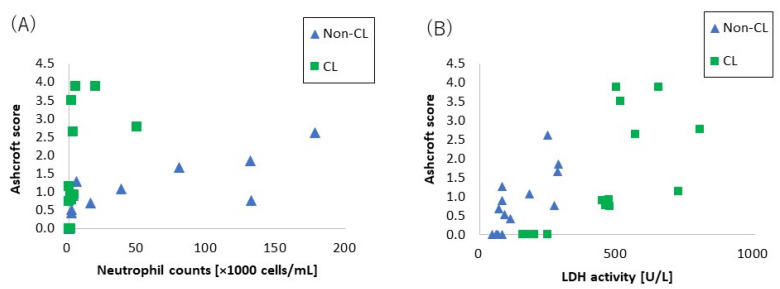
The relationship between lung fibrosis and lung injury. (**A**) The relationship between lung fibrosis and neutrophil counts. (**B**) The relationship between lung fibrosis and released LDH activity. Neutrophil counts in BALF showed a biphasic upward trend between Non-CL-PAA and CL-PAA, while LDH, an indicator of cytotoxicity, showed a nearly linear increase in response to fibrosis.

Figure 7 shows the relationship between the lung fibrosis and lung injury. Neutrophil counts in the BALF showed a biphasic upward trend between non-crosslinked and crosslinked PAA (Figure 7A), while LDH, an indicator of cytotoxicity, showed a nearly linear increase in response to fibrosis (Figure 7B).

### 2.6. Gene Expression Analysis

Table 1 and Table 2 show the number of genes, among the 20,174 genes examined by cDNA microarray, sorted by the fold change of mRNA expression levels in the lung tissue at 1 month in the 1.0 mg exposure group of CL-PAA compared with that of Non-CL-PAA. A total 266 genes were more than 1.5-fold upregulated; the top 20 of them are shown in Table 1. The gene-related fibroblast cells in the top 10 of those genes are shown in Table 2. The result of KEGG pathway revealed that differentially expressed upregulated genes were significantly enriched in 7 pathways (Table 3), including the “Apoptosis” and “PI3K-Akt signaling” pathway.

## 3. Discussion

In the present study, Non-CL-PAA and CL-PAA were intratracheally instilled into rats, and CL-PAA significantly caused lung fibrosis compared with Non-CL-PAA. We discussed (1) inflammogenicity and fibrogenicity induced by both types of PAA, (2) pathophysiology accompanied with the crosslinked type, and (3) factors related to the crosslinking structure of PAA in the progress of fibrosis.

**Figure 8:**
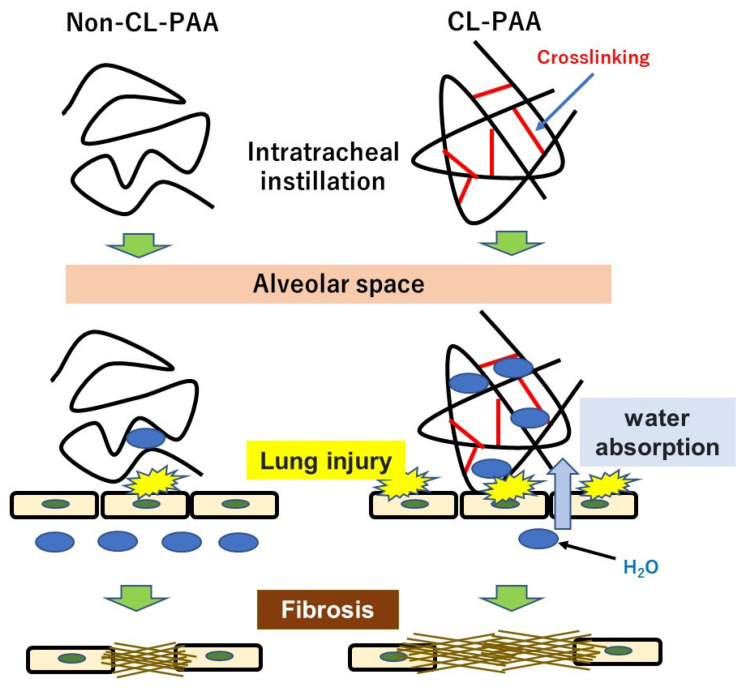
Schematic overview of the study. CL-PAA cause osmotic pressure changes due to water absorption, and this difference in water absorption by PAAs may have influenced the difference in fibrosis via cell injury.

(1) Inflammogenicity and fibrogenicity induced by both types of PAA. In the present study, both Non-CL-PAA and CL-PAA induced severe lung inflammation in the acute phase but it was transient. In addition, fibrosis occurred to a certain degree in both of the PAAs. In the past, persistent lung inflammation was considered to lead to irreversible fibrosis in lung exposed to inhaled chemicals. In the case of silica and asbestos, which have high pulmonary toxicity, there was persistent inflammation that progressed to fibrosis in rat lung up to 6–12 months after exposure [10,13,14,15]. On the other hand, inhaled chemicals such as titanium dioxide (TiO_2_) and zinc oxide (ZnO), which have low pulmonary toxicity, show acute-phase lung inflammation and lung injury, but inflammation is recovered from within 1 week to 1 month after exposure and does not progress to fibrosis [16,17,18]. In the present study, lung inflammation was caused for about 1 week, and CINC, a neutrophil chemokine, increased significantly in both PAAs to 1 week in a dose-dependent manner. Total protein and LDH in the BALF (tissue injury indicators) and HO-1 in the lung tissue (oxidative stress indicator) were also significantly increased in the exposed groups until 1 month after exposure.
  Considering that persistent inflammation is associated with the potential for lung disorder, it is speculated that the lung disorder potential of both PAAs is not high, based on transient inflammation and inflammation-associated cytokine production by both PAAs. However, although the inflammation by PAAs ended at the acute phase, it was accompanied by fibrosis, and we considered that the acute inflammation rapidly transitioned to the next stage, fibrosis—that is, it progressed to an irreversible lesion.
  Fibrosis after acute inflammation has been observed in animal exposure studies and clinical cases; it is often observed in LPS exposure models that induce acute inflammation and in ARDS that presents with acute respiratory failure. The clinical presentation of ARDS is known to cause lung injury of the alveolar epithelium due to inflammation followed by fibrosis from repair processes [19]. Animal models of acute respiratory failure caused by LPS or bleomycin also show severe lung inflammation that ends in 1–2 weeks, followed by fibrosis [20,21]. Although the persistent inflammation by both PAAs was not observed for long periods, the inflammations did cause fibrosis. Similarly, in other reports of PAA, the inflammation is followed promptly by fibrosis [7,10]. Although asbestosis usually causes fibrosis after 10 years of exposure in clinical cases, PAA causes rapid fibrosis in 1 to 2 years in the clinical cases of workers working with it. The present study is clinically consistent with PAA having fibrotic potential.
  In the present study, both exposures to PAA caused irreversible fibrosis from 1 month after intratracheal instillation. Proliferation of fibroblasts or deposition of collagen in the lung interstitium has been observed from about 1 month after pharyngeal aspiration exposure of asbestos or MWCNTs in mice [14]. An intratracheal instillation in mice using the organic substance polyhexamethylene guanidine phosphate (PHMG-*p*) also showed collagen deposition as well as lung inflammation from 2 weeks after intratracheal instillation [22]. Considering these reports, the fibrosis observed in the present study after exposure to Non-CL-PAA and CL-PAA suggests that both polymers have fibrogenic potential.
(2) Pathophysiology accompanied with the crosslinked type. The level of crosslinking of CL-PAA in the present study was below 0.1%, which is commonly used in industrial applications for its thickening and water absorbency properties, and the fibrogenicity of CL-PAA in the lung with the level of crosslinking in industrial applications has been evaluated. In the present study, the CL-PAA caused more fibrosis of the lungs than did the Non-CL-PAA. To investigate the difference in fibrosis progression between the CL-PAA and Non-CL-PAA, we performed a microarray analysis using lung tissue one month after exposure, which is considered to be the fibrosis transition period. Compared with Non-CL-PAA, the expression of elastin, which constitutes elastic fibers, and the collagen 1a1 gene, which is involved in the fibrosis, was higher in CL-PAA. Increased elastin gene and collagen 1a1 gene expression in fibrotic lesions have been reported in a bleomycin-induced pulmonary fibrosis model [23,24], suggesting that increased elastin as well as collagen was involved in the fibrosis of the lung histopathology findings of CL-PAA.
  In the CL-PAA with strongly induced fibrosis, the LDH in the BALF was found to be higher than in Non-CL-PAA, suggesting that CL-PAA causes greater cellular injury to the constituent cells of the lung. Several reports have recognized that stronger lung injury leads to the development of fibrosis: in inhalation exposure and intratracheal instillation of MWCNTs in mice, it was reported that the increase in the LDH in BALF and the deposition and fibrosis levels of MWCNTs showed similar tendencies to increase [14,25]. In the present study, the relationship between LDH and fibrosis in the BALF with both PAAs showed an almost linear correlation between LDH and fibrosis (Figure 7A). On the other hand, a correlation was observed between the number of neutrophils in the BALF and fibrosis, but not as linear as the correlation between LDH and fibrosis (Figure 7B). It is likely that lung injury has a more direct effect on the fibrosis development of PAA than does inflammatory cell infiltration.
  As for gene expression involved in lung injury including cell death, KEGG pathway analysis in genes that are more upregulated in CL-PAA showed genes related to apoptosis, including apoptosis and the Phosphoinositide 3-kinase-AKT (PI3K-AKT) signaling pathway, and it is reported that these pathways are involved in fibrosis. In models of idiopathic pulmonary fibrosis and bleomycin-induced pulmonary fibrosis, it has been reported that the expression of genes related to the PI3K-AKT pathway is elevated and involved in the development of fibrosis in the lung [26,27]. Although apoptosis is necessary for normal repair to eliminate inflammatory cells and proliferating fibroblasts that collect in the alveolar walls and alveolar space during lung injury [28], it has been reported that excessive apoptosis is involved in the pathogenesis of ARDS, which is a transition to fibrosis [26]. Considering the increased expression of collagen genes and the enhancement of the apoptotic pathways, including the PI3K-AKT pathway in lung tissue due to lung injury, the progression of fibrosis in CL-PAA may be related to lung injury.
(3) Factors related to the crosslinking structure of PAA in the progress of fibrosis. The molecular weights of Non-CL-PAA and CL-PAA in this study were 7.53 × 10^5^ g/mol and 7.65 × 10^5^ g/mol, respectively, and the radius of gyration (Rg) indicated that the radii of polymer particles in solution were 74.9 nm and 68.7 nm, respectively. Since both PAAs have similar molecular weights, we considered that the crosslinking structure was responsible for the enhancement of fibrosis by the PAA. In general, the thickening properties of PAA change with increasing molecular weight [8]. It has been reported that there is an optimal viscosity for sputum clearance [29,30]. The thickening that comes with increasing molecular weight may affect the delay in clearance of the PAA from the lungs. In the present study, the rat lungs at dissection 3 days to 1 month after intratracheal instillation of PAA, especially in the 1.0 mg exposure group, were not expanded, and BALF was not recovered sufficiently even when saline was infused into the lung (Figure 1B). Such poor recovery of BALF may reflect the result of a collapsed lung, and a collapsed lung may affect the delay in clearance of PAA. On the other hand, the presence of a crosslinked structure makes a high water-absorbency property and low viscosity when the molecular weight of both PAAs are the same level [31,32]. In other words, CL-PAA incorporates water molecules into the polymer’s strong braided structure by crosslinking in the lung, while Non-CL-PAA has a weak retention of water molecules due to its lack of a strong network structure. It is possible that the water-absorbing property of the CL-PAA changes the osmotic pressure in the lungs by absorbing water molecules in vivo, causing cytotoxicity. In vitro studies using polymeric nanoparticles have shown that cytotoxicity increases with elevation in the osmotic pressure of the culture medium. When the biodegradable polymer Poly(lactideco-glycide) was exposed to macrophages (RAW cells) and alveolar epithelial cells (A549 cells), it caused cell death as the osmotic pressure of the medium increased, and a correlation was observed between the increase in osmotic pressure and cell death [33]. Since PAAs also cause osmotic pressure changes due to water absorption, this difference in water absorption by PAAs may have influenced the difference in fibrosis via cell injury (Figure 8).

In the present study, the maximum intratracheal instillation dose was set at 1.0 mg. These doses were set to not reach overdose, assuming human exposure. Regarding toner particles as poorly soluble particles, it has been reported that lung damage due to overdose in addition to toxicity of the substance itself occurs when the amount of deposition in the lungs exceeds 1 to 3 mg [34,35]. We previously examined the biopersistence of TiO_2_ nanoparticles with low toxicity among nanomaterials in rat lung in an intratracheal instillation study, and the clearance of TiO_2_ nanoparticles in the rat lung accompanied by neutrophil inflammation began to delay at doses exceeding 1 mg/rat [36]. We considered that pulmonary inflammation by a high dose of more than 1.0 mg/rat may be induced by the excessive dose of material, not the toxicity of the material itself.

Intratracheal instillation studies can be helpful for approximating the hazardous effects of inhalable chemicals; however, a limitation of this study is that its exposure route was not physiological and bolus effects were caused. Despite the instillation of PAA of a respirable size, it is not the same as in inhalation studies in terms of the distribution, clearance, and retention of materials.

## 4. Materials and Methods

### 4.1. Sample Polymer

**Figure A1:**
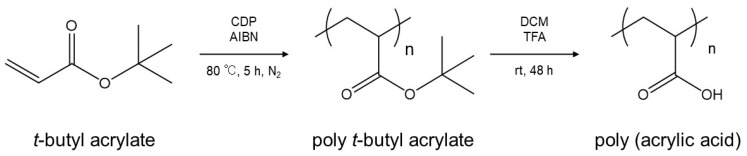
Overview for synthesis of Non-CL-PAA. Reversible Addition/Fragmentation Chain Transfer Polymerization were conducted using 4-Cyano-4-[(dodecylsulfanylthiocarbonyl) sulfanyl]pentanoic acid (CDP, Sigma Aldrich), α,α′-Azobisisobutyronitrile(AIBN, KANTO CHEMICAL CO., INC.), and t-butyl acrylate. The dissolved solution was transferred to a Schlenk tube and bubbled with nitrogen for 30 min. The reaction was then carried out in an oil bath at 80 °C for 5 h, and purified by re-precipitation in a mixture of water and methanol (2:8) and filtration. The polymer was dissolved in THF and dried to obtain poly t-Butyl acrylate. The resulting poly t-Butyl acrylate was dissolved in dichloromethane (DCM), trifluoroacetic acid (TFA) was added, and the reaction was carried out at room temperature for 48 h to deprotect the t-butyl group to obtain PAA. Hexane was added to the reaction solution to precipitate the PAA, and the solvent was removed by filtration. The resulting polymer was then dissolved in methanol, dialyzed, purified, and lyophilized with 1,4-dioxane to obtain Non-CL-PAA.

A sample of CL-PAA was purchased from Sigma-Aldrich, St. Louis, MO, USA. CL-PAA is a white, easily scattered powder, with a specific gravity of 1.2. The degree of crosslinking of a polymer is generally expressed as the weight % of the crosslinker in the raw material. The degree of crosslinking of our CL-PAA sample was 0.1% or less. A sample of Non-CL-PAA was synthesized by the polymerization of an acrylic acid monomer without a crosslinking agent. Briefly, Reversible Addition/Fragmentation Chain Transfer Polymerization were conducted using 4-Cyano-4-[(dodecylsulfanylthiocarbonyl) sulfanyl]pentanoic acid (CDP, Sigma Aldrich, St. Louis, MO, USA), α,α′-Azobisisobutyronitrile (AIBN, KANTO CHEMICAL CO., INC., Tokyo, Japan), and t-butyl acrylate. Next, Deprotection of the t-butyl group of the resulting poly t-Butyl acrylate was performed to obtain Non-CL-PAA without using a crosslinking agent (Figure A1). Non-CL-PAA and the CL-PAA were mixed with distilled water and slowly stirred for 40 min (Mag-Mixer MF820 or MD300, Yamato Scientific co., Ltd., Tokyo, Japan). The weight average molecular weight (MW), number average molecular weight (Mn), and poly dispersity index (PDI) of the polymer and the radius of gyration (Rg) were measured by gel permeation chromatography (GPC) (a Prominence 501 system (SHOKO SCIENCE, Kanagawa, Japan) coupled with a multi-angle static light scattering (MALS) detector (Dawn-Heleos-II, Wyatt Technology Europe GmbH, Dernbach, Rheinland-Pfalz, Germany) using GF-7MHQ (Showa Denko K.K., Tokyo, Japan) with 0.1 M carbonate-bicarbonate buffer as the eluent [37,38]. The viscosity of the PAA suspensions was measured as 25 °C using a viscometer (VISCO lab 4000, Cambridge Viscosity, Inc., Boston, MA, USA) with a temperature controller (F12-MB, JULABO GmbH, Seelbach, Germany).

**Figure 9:**
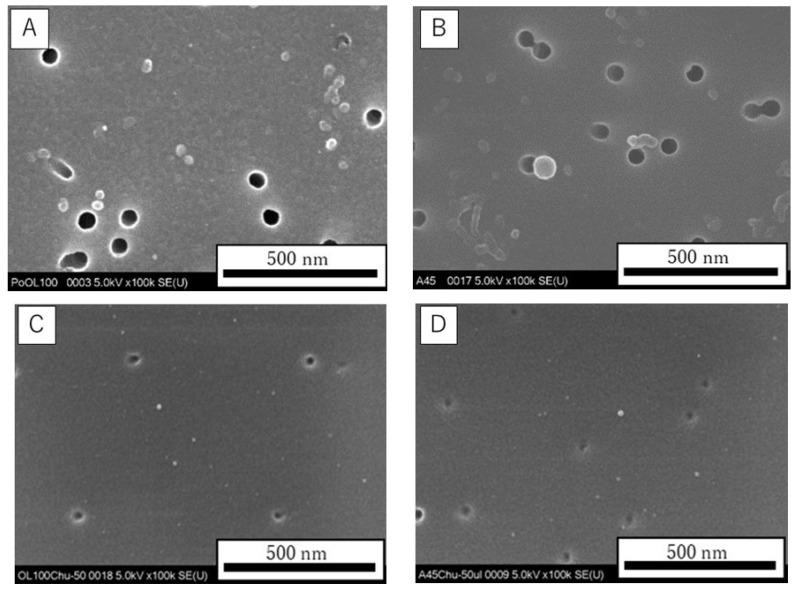
SEM images of the polymers used in the present study. Suspension of Non-CL-PAA and CL-PAA in the molecular dispersion (**A**,**B**), and the distilled water (**C**,**D**), respectively (internal scale bar = 500 nm).

The fundamental characteristics of Non-CL-PAA and CL-PAA are summarized in Table 4. The Non-CL-PAA and CL-PAA used in our study had weight average molecular weights (MW) of 7.53 × 10^5^ g/mol and 7.65 × 10^5^ g/mol, respectively. In the molecular dispersion, the polymers were dissolved in 0.1 M carbonate–bicarbonate buffer; then, the solutions were alkalized with 2N NaOH and neutralized with 1N HCl. Figure 9 shows the scanning electron microscopy (SEM) images obtained using a HITACHI S-4500 (Hitachi, Ltd., Tokyo, Japan) of the dispersed Non-CL-PAA and CL-PAA in the molecular dispersion (Figure 9A,B) and the distilled water (Figure 9C,D), respectively.

### 4.2. Animals

Male Fischer 344 rats (8 weeks old) (Charles River Laboratories International, Inc., Kanagawa, Japan) were acclimated for 4 weeks in the Laboratory Animal Research Center of the University of Occupational and Environmental Health, Japan with free access to a commercial diet and water. All procedures and animal handling were performed according to the guidelines described in the Japanese Guide for the Care and Use of Laboratory Animals as approved by the Animal Care and Use Committee, University of Occupational and Environmental Health, Japan (animal studies ethics clearance proposal number: AE17-009).

### 4.3. Intratracheal Instillation

Doses of 0.2 mg (0.8 mg/kg BW) and 1.0 mg (4.0 mg/kg BW) of Non-CL-PAA and CL-PAA suspended in 0.4 mL distilled water were administered to the lungs of rats (12 weeks old) in single intratracheal instillations. Rats were intratracheally instilled under anesthesia by sevoflurane (Pfizer Japan, Tokyo, Japan) inhalation. Briefly, a laryngeal extension was performed using a laryngoscope blade (MAC1, Rudolf Riester GmbH, Jungingen, Germany), an animal feeding needle (KN-348, Natsume Seisakusho Co., Ltd., Tokyo, Japan) was inserted directly into the trachea, and the suspension was manually injected. Then, 3 mL of air twice with a syringe from the animal feeding needle was inserted into the trachea. The rats were then allowed to awaken spontaneously and were observed periodically. Single intratracheal instillations of 0.2 mg and 1.0 mg of Non-CL-PAA and CL-PAA were performed at different times, and the single intratracheal instillations were conducted a total of two times. The control group received distilled water, and a control group was established for each intratracheal instillation. Dosages of 0.2 and 1.0 mg/rat were used in the intratracheal instillation of the PAAs. This maximum dose was set assuming human exposure and to avoid overload in the lung [10].

### 4.4. Animals Following Intratracheal Instillation

There were 5 rats in each exposure and control group at each time point. Animals were dissected at 3 days, 1 week, 1 month, 3 months, and 6 months after intratracheal instillation under anesthesia by isoflurane (Pfizer Japan, Tokyo, Japan) inhalation. Body and lung weights were measured; then, at autopsy, blood was removed from the abdominal aorta and the lungs were perfused with normal saline. The right lungs were repeatedly inflated with normal saline under a pressure of 20 cm H_2_O, following fluid recovery two times, while the left main bronchus was clamped. Between 7 and 14 mL of the recovered fluid (BALF) was collected in collection tubes by free fall, and then the right and left lungs were divided. The homogenized third lobes of the right lungs were used for HO-1 and cDNA microarray after recovery of BALF. The left lungs were inflated and fixed by 10% formaldehyde under a pressure of 25 cm H_2_O for use in histopathological evaluation.

### 4.5. Cytospin Analysis of Inflammatory Cells and Measurement of Inflammation Related Markers in BALF

BALF was centrifuged at 400× *g* at 4 °C for 15 min, and the supernatant was transferred to a new tube for measurement of total protein, lactate dehydrogenase (LDH), and cytokines. The pellets were washed by suspension with polymorphonuclear leukocyte (PMN) Buffer (137.9 mM NaCl, 2.7 mM KCl, 8.2 mM Na_2_HPO_4_, 1.5 mM KH_2_PO_4_, and 5.6 mM C_6_H_12_O_6_) and centrifuged at 400× *g* at 4 °C for 15 min. After removal of the supernatant, the pellets were resuspended with 1 mL of PMN (polymorphonuclear leukocyte) Buffer. The number of cells in the BALF was counted by ADAM-MC (AR BROWN CO., LTD, Tokyo, Japan); the cells were splashed on a slide glass using cytospin, and fixed and stained with Diff-Quik (Sysmex CO., Kobe, Hyogo, Japan), after which the number of neutrophils and alveolar macrophages were counted by microscopic observation. The released LDH and ALP activity in the BALF supernatant was measured by a Cytotoxicity Detection KitPLUS (LDH) (Roche Diagnostics GmbH, Mannheim, Nordrhein-Westfalen, Germany) according to the manufacturer’s instructions. LDH activity was estimated using a standard curve obtained from known concentrations of recombinant LDH from rabbit muscle (Oriental Yeast Co., ltd., Tokyo, Japan). The concentration of protein in the supernatant of the BALF was determined by a Pierce™ 660 nm Protein Assay (Thermo Scientific Inc., Rockford, IL, USA).

### 4.6. Measurement of Chemokines in BALF and HO-1 in Lung Tissue

Concentrations of CINC-1 and CINC-2 in the BALF were measured by ELISA kits #RCN100 and #RCN200 (R&D Systems, Minneapolis, MN, USA), respectively. All measurements were performed according to the manufacturer’s instructions. The third lobes of the right lungs were homogenized with a T-PER tissue protein extraction reagent (Thermo Scientific Inc., Rockford, IL, USA) including protein inhibitor cocktails (P8340, Sigma-Aldrich, St. Louis, MO, USA) and cOmplete Mini (Roche Diagnostics GmbH, Mannheim, Nordrhein-Westfalen, Germany), and then centrifuged (20,400× *g* at 4 °C for 10 min). The protein concentration in the supernatant was measured by a Pierce 660 nm Protein Assay Reagent (Thermo Scientific Inc., Rockford, IL, USA), using bovine serum albumin as a standard. The results of the HO-1 measurements with ELISA kit, ADI-EKS-810A (Enzo Life Sciences, Farmingdale, NY, USA) were corrected by the protein concentration in the supernatant to calculate the final HO-1 concentration in the lung tissue.

### 4.7. Total RNA Extraction

The third lobes of the right lungs (*n* = 5 per group per time point) were homogenized while using a QIAzol lysis reagent with a TissueRupotor (Qiagen, Hilden, Germany). Total RNA from the homogenates was extracted using a miRNeasy Mini Kit (Qiagen, Hilden, Germany) following the manufacturer’s instructions. RNA was quantified using a NanoDrop 2000 spectrophotometer (Thermo Fisher Scientific Inc., Waltham, MA, USA), and the quality of the samples was analyzed by a Bioanalyzer 2100 (Agilent Technologies, Santa Clara, CA, USA).

### 4.8. Microarray Analysis

We used a three-dimensional (3D)-Gene Rat Oligo Chips 20K (version 1.1) (Toray Industries, Tokyo, Japan), which could mount 20,174 genes, for the DNA microarray analysis. The total RNA extracted from the lungs of the five rats in the high dose Non-CL-PAA group was mixed in equal amounts to make one sample, which was then amplified by the use of an Amino Allyl MessageAmp II aRNA Amplification Kit (Ambion, Inc., Austin, CA, USA). The CL-PAA of the high-dose group was treated in the same manner.

The antisense RNA (aRNA) were labeled with Cy5, using Amersham Cy5 Mono-Reactive Dye (GE Healthcare, Buckinghamshire, UK), and the labeled aRNA were hybridized at 37 °C for 16 h. The hybridization was performed according to the supplier’s protocols. The chips were washed and dried, then scanned in an ozone-free environment using a 3D-Gene Scanner 3000 (Toray Industries, Tokyo, Japan) and analyzed by use of 3D-Gene Extraction Software (Toray Industries, Tokyo, Japan). Each raw signal intensity was divided by the signal intensities of the beta-actin (ACTB) and glyceraldehyde-3-phosphate dehydrogenase (GAPDH), which are known internal standards in the samples, to compare the gene expression levels of Non-CL-PAA and CL-PAA, respectively. We analyzed the relationship genes that were found to be elevated in CL-PAA using the above microarray data for Kyoto Encyclopedia of Genes and Genomes (KEGG) pathway analysis via the Database for Annotation, Visualization and Integrated Discovery (DAVID) version 2021 (https://david.ncifcrf.gov/home.jsp) (accessed on 29 July 2022).

### 4.9. Histopathology

Formaldehyde-fixed lung tissue was embedded in paraffin, sectioned at a thickness of 4 μm, and then stained with hematoxylin and eosin (HE) and Masson trichrome (MT) staining. The lung inflammation and fibrosis were examined using the inflammatory cell infiltration score [39] and the Ashcroft score [40,41], respectively, as in previous reports [10,34]. Briefly, the inflammatory cell infiltration score was obtained by scoring the degree of inflammatory cell infiltration in lung tissue as follows: none (0), minimal (0.5), mild (1), moderate (2), or severe (3). Lung fibrosis was assessed by scoring the lung histopathological findings on a scale of 0 to 8, using the Modified Scale of Ashcroft score, and the mean and standard deviation were calculated in each group.

### 4.10. Statistical Analysis

Statistical analysis was carried out using IBM^®^ SPSS^®^ software (IBM Corporation, Chicago, IL, USA). *p*-values < 0.05 were considered statistically significant. Dunnett’s tests and Tukey’s honestly significant difference test were used appropriately to detect individual differences between those exposed to the crosslinked polyacrylate samples and the controls.

## 5. Conclusions

We conducted intratracheal instillations using Non-CL-PAA and CL-PAA in rats and evaluated the pulmonary pathology. Both PAAs induced lung inflammation and fibrosis, and CL-PAA with a degree of crosslinking within 0.1% was found to have stronger fibrotic potential and lung injury than Non-CL-PAA, suggesting that the crosslinking structure in PAA, which is used in crosslinking levels in industrial applications, may contribute to the progression of the fibrogenicity of PAA.

## Figures and Tables

**Table 1:** Upregulating of the top 20 genes in lung exposed to CL-PAA.

Gene Symbol	Gene Description	Fold Change (ACTB)	Fold Change (GAPDH)
Mt3	metallothionein 3	5.6	8.0
AABR07056026.1	-	3.1	4.4
Olr1523	olfactory receptor 1523	3.0	4.3
Hbq1b	hemoglobin, theta 1B	2.8	4.1
Mt2A	metallothionein 2A	2.8	4.1
Rab24	RAB24, member RAS oncogene family	2.8	4.0
Tuba3b	tubulin, alpha 3B	2.8	4.0
Nr1d1	nuclear receptor subfamily 1, group D, member 1	2.6	3.8
Igh-6	immunoglobulin heavy chain 6	2.6	3.7
RGD1565617	similar to Ig variable region, light chain	2.6	3.7
Ttll11	tubulin tyrosine ligase like11	2.5	3.6
Atp13a2	ATPase 13A2	2.4	3.4
Sec14l1	SEC14-like lipid binding 1	2.3	3.3
Igsf8	immunoglobulin superfamily, member 8	2.2	3.1
Egfl7	EGF-like-domain, multiple 7	2.2	3.1
Fau	Finkel–Biskis–Reilly murine sarcoma virus (FBR-MuSV) ubiquitously expressed	2.1	3.0
Eln	elastin	2.1	3.0
Ep300	E1A binding protein p300	2.0	2.9
Arl2	ADP-ribosylation factor like GTPase 2	2.0	2.9
Otop2	otopetrin 2	1.5	2.1

**Table 2:** The top 10 genes related to fibroblast cell in lung exposed to CL-PAA.

Gene Symbol	Gene Description	Fold Change (ACTB)	Fold Change (GAPDH)
Eln	elastin	2.1	3.0
Akt1	v-akt murine thymoma viral oncogene homolog 1	1.8	2.6
Ccm2l	CCM2-like scaffolding protein	1.7	2.4
Mif	macrophage migration inhibitory factor (glycosylation-inhibiting factor)	1.6	2.3
Parp10	poly (ADP-ribose) polymerase family, member 10	1.6	2.3
Pdgfrb	platelet derived growth factor receptor beta	1.5	2.1
Col1a1	collagen, type I, alpha 1	1.4	2.1
Fgfr4	fibroblast growth factor receptor 4	1.4	2.0
Ctgf	connective tissue growth factor	1.4	2.0
Hyal2	hyaluronoglucosaminidase 2	1.4	2.0

**Table 3:** The result of KEGG pathway using more than 1.5-fold upregulated genes in lung exposed to CL-PAA.

Terms of KEGG Pathway	Gene Counts	%	*p*-Value
Salmonella infection	10	5.08	0.0012
Alzheimer disease	12	6.09	0.0021
Tuberculosis	8	4.06	0.0022
Apoptosis	7	3.55	0.0027
Fluid shear stress and atherosclerosis	7	3.55	0.0045
PI3K-Akt signaling pathway	10	5.08	0.0088
Sphingolipid signaling pathway	6	3.05	0.0089

**Table 4:** Physiochemical characterization of the polymer used in the present study.

Physiochemical Characterization	Non-Crosslinked Polyacrylic Acid	Crosslinked Polyacrylic Acid
Structural formula	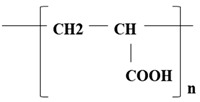	
Weight average molecular weight (MW)	7.53 × 10^5^ g/mol	7.65 × 10^5^ g/mol
Number average molecular weight (Mn)	6.10 × 10^5^ g/mol	4.14 × 10^5^ g/mol
Poly dispersity index (PDI)	1.24	1.85
Degree of crosslinking	None	~0.1%
Viscosity (mPa·s) at 25 °C	3.039 mPa·s	2.383 mPa·s
radius of gyration (Rg)	74.9 nm	68.7 nm

## Data Availability

Not applicable.
